# CUEDC2 ablation enhances the efficacy of mesenchymal stem cells in ameliorating cerebral ischemia/reperfusion insult

**DOI:** 10.18632/aging.202394

**Published:** 2021-01-20

**Authors:** Yan Huang, Xia Xiao, Han Xiao, Zhiping Hu, Fengbo Tan

**Affiliations:** 1 National Health Commission Key Laboratory of Birth Defects Research, Prevention, and Treatment, Hunan Provincial Maternal and Child Health Care Hospital, Changsha 410008, Hunan, P.R. China; 2 Key Laboratory of Protein Chemistry and Developmental Biology of Ministry of Education, College of Life Sciences, Hunan Normal University, Changsha 410081, Hunan, P.R. China; 3 Hunan Provincial Key Laboratory of Neurorestoration, Changsha 410003, Hunan, P.R. China; 4 Department of Neurology, The Second Xiangya Hospital, Central South University, Changsha 410011, Hunan, P.R. China; 5 Department of Gastrointestinal Surgery, Xiangya Hospital, Central South University, Changsha 410008, Hunan, P.R. China

**Keywords:** MSCs, CUEDC2, OGD/R, MCAO

## Abstract

Mesenchymal stem cell (MSC) therapy has been reported to be a promising therapeutic option for cerebral ischemia/reperfusion (I/R) insult. However, the poor survival rate of engrafted MSCs under unfavorable cerebral I/R-induced microenvironment inhibits their efficiency during clinical application. CUE domain-containing 2(CUECD2) exhibits its protective role on cardiomyocytes by mediating the antioxidant capacity. Our study explored the functional role of CUEDC2 in cerebral I/R challenge and determined whether CUECD2-modified MSCs could improve the efficacy of treatment of the insulted neurons. We also evaluated the possible mechanisms involved in cerebral I/R condition. Cerebral I/R stimulation suppressed CUEDC2 levels in brain tissues and neurons. siRNA-CUEDC2 in neurons significantly inhibited cerebral I/R-induced apoptosis and oxidative stress levels *in* *vitro*. Moreover, siRNA-CUEDC2 in the MSCs group remarkably enhanced the therapeutic efficacies in cerebral I/R-induced neuron injury and brain tissue impairment when compared to the non-genetic MSCs treatment group. At the molecular level, siRNA-CUEDC2 in MSCs markedly enhanced its antioxidant and anti-inflammatory effect in co-cultured neurons by upregulating glutathione peroxidase 1 (GPX1) expression levels while suppressing NF-kB activation. These findings provide a novel strategy for the utilization of MSCs to promote cerebral ischemic stroke outcomes.

## INTRODUCTION

Cerebral ischemic stroke is a life-threatening neurological disease that is characterized by a sudden disruption in the supply of cerebral blood flow [1]. Thrombolysis therapy is the gold standard therapeutic option for cerebral ischemia stroke [2]. However, the subsequent reperfusion phase for recovering cerebral blood flow in ischemic brain tissues could eventually result in irreversible brain damage. Multiple and complex molecular mechanisms, including excessive oxidative stress and inflammatory responses, calcium overload as well as apoptotic cell death, are involved in the progression and pathogenesis of cerebral I/R-induced injury [3, 4]. The clinical benefits of MSCs therapy as a promising strategy for clinical application in ischemic stroke have been reported [5]. Transplanted MSCs can repair or suppress neuro-degradation by inhibiting cell death and secreting a series of growth factors, as well as anti-inflammatory cytokines in the cerebral I/R damaged brain tissues [6]. However, a significant number of the transplanted MSCs undergo cell death that is attributed to the unfavorable microenvironment induced by excessive oxidative stress following I/R. Cell death affects the therapeutic efficacy of the engrafted MSCs [7–9]. Therefore, it is essential to optimize the clinical efficacy of MSCs in the treatment of cerebral I/R.

Several feasible strategies for enhancing the clinical efficacy of MSCs in the management of cerebral I/R-induced insult have been proposed. They include the regulation of inflammatory-immune responses in the cerebral I/R environment [6], hypoxia preconditioning [10] and genetic modification of MSCs before transplantation [11, 12]. CUEDC2 is a novel protein-coding gene with a molecular weight of approximately 32 kDa [13]. CUEDC2 is associated with the progesterone receptor (PR) and is involved in the ubiquitination and degradation of PR in breast cancer [14]. CUEDC2 is expressed in different tissues or organs, such as the heart, brain and liver as well as in various cancer types [15–17]. Li et al. reported that downregulated CUEDC2 expression in glioma and glioma cell lines led to the promotion of tumorigenicity and proliferation of tumor cells through the activation of STAT3 and nuclear factor kappa B (NF-kB) pathway [18]. Contrastingly, Zhang et al. demonstrated a high expression of CUEDC2 in myeloid leukemia that enhanced cell apoptosis and inhibited imatinib resistance by suppressing NF-kB activation [19]. Furthermore, CUEDC2 has been confirmed to be a critical modulator of bone formation and osteoblast differentiation through the SOCS3-STAT3 signaling pathway [20]. Although previous studies have established the multiple functional roles of CUEDC2 in tumorigenesis, inflammation and bone information, the effect of CUEDC2 on neuronal cells following cerebral I/R insult have not been elucidated. More importantly, the significance of CUEDC2-modified MSCs on cerebral I/R-induced injury has not been evaluated. A recent study demonstrated a novel protective role of CUECD2 in cardiomyocytes I/R injury [21]. CUEDC2 silencing upregulated the antioxidant capacity in cardiomyocytes, and enhanced the reactive oxygen species (ROS) scavenging ability by regulating the redox-associated pathway and the stability of glutathione peroxidase 1 (GPX1) in the cardiomyocytes I/R environment. Therefore, we hypothesized that CUEDC2 might also participate in cerebral I/R injury.

In this study, we determined the possible functional roles of CUEDC2 in cerebral I/R-induced injury. Moreover, we evaluated whether CUECD2-modified MSCs could improve therapeutic effects in the injured neurons and the potential mechanisms underlying cerebral I/R recovery.

## RESULTS

### CUEDC2 in the rat brain is expressed upon cerebral I/R-induced insult

**Figure 1:**
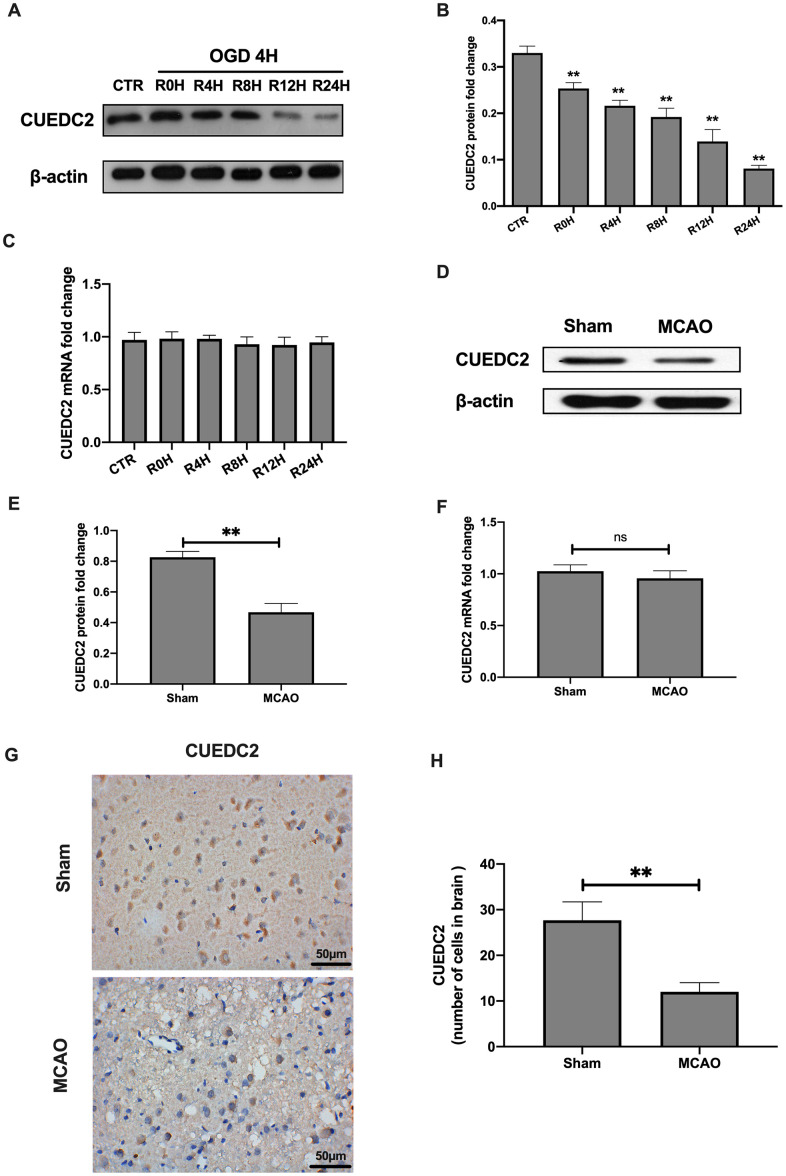
**Cerebral I/R induced the downregulation of CUEDC2 expression in brain tissues.** (**A**–**C**) Protein and mRNA expression of CUEDC2 in neurons as detected by western blotting and PCR assays. (**D**–**F**) Protein and mRNA expression of CUEDC2 in brain tissues as assayed by western blotting and PCR. (**G**, **H**) Protein expression of CUEDC2 in brain tissues as assayed by immunohistochemical staining. Scale bar = 50 μm. CTR, control; CUEDC2: CUE domain-containing 2; OGD/R: oxygen-glucose deprivation (4 hours) and reperfusion (0,4,8,12, and 24 hours); MCAO, middle cerebral artery occlusion. All data are presented as the mean value ± SD. *p<0.05, **p<0.01; compared to the control group, sham group.

The protein levels of CUEDC2 were found to be gradually decreased in the neurons at 4-hours of OGD/R and at different reperfusion time points (Figure 1A–1B). This degradation trend was lowest at 24-hours of reperfusion time points. However, there were no significant differences in the mRNA expression levels of CUEDC2 in neurons after OGD at various time points between the reperfusion insult group and the control group (Figure 1C). Consistent with neuronal findings, protein expression levels of CUEDC2 in the rat brain tissues of the MCAO group were significantly low when compared to the sham group. Moreover, the mRNA expression was not obviously changed and differences in either the sham group or the MCAO group (Figure 1D–1F). Immunohistochemical-staining showed that CUEDC2 expression was suppressed in the MCAO group when compared to the sham group (Figure 1G–1H). These results indicate that cerebral I/R stimulation suppressed CUEDC2 levels in the rat brain tissues and neuron cells.

### Effects of CUEDC2 on cerebral I/R-induced neuron insult

**Figure 2:**
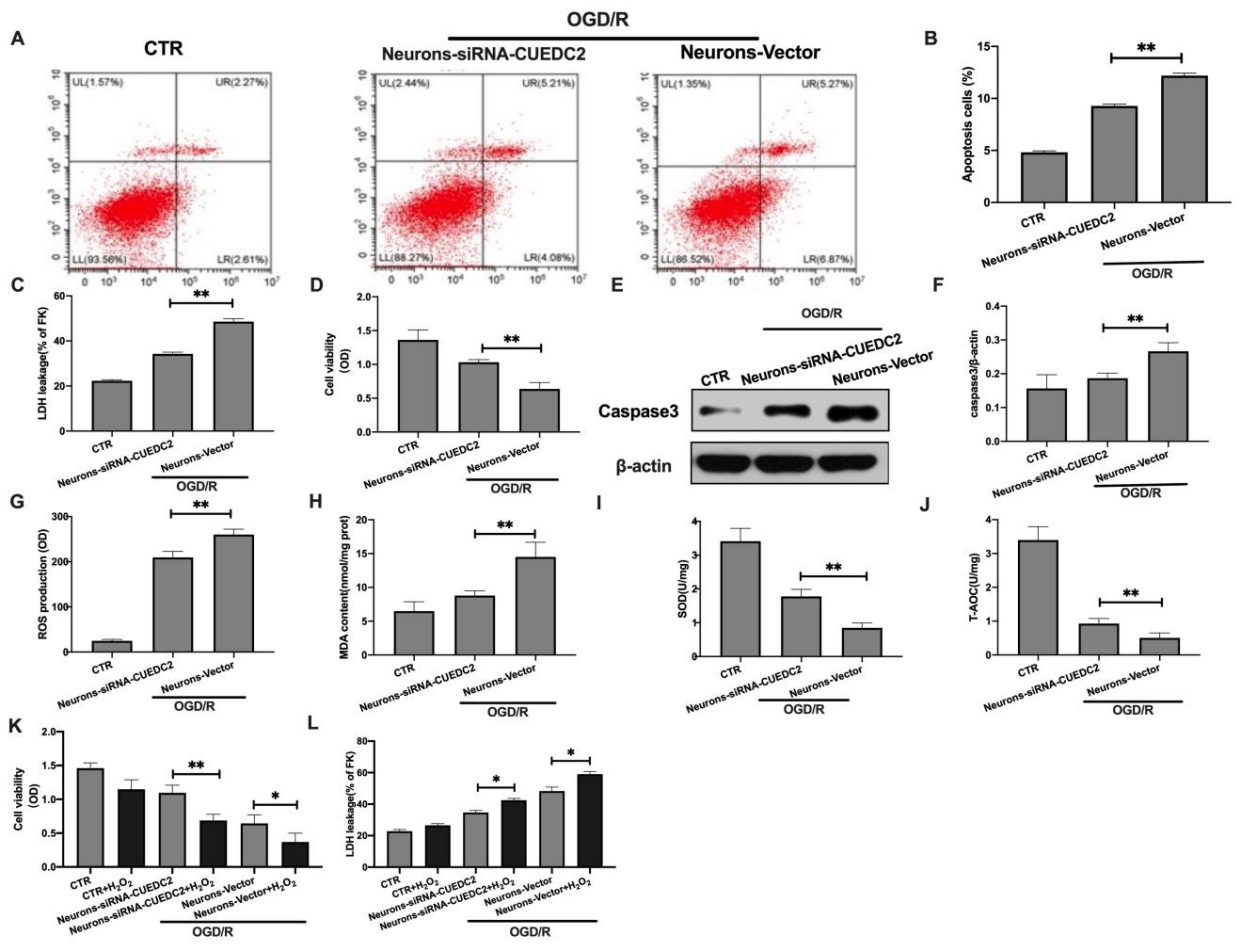
**Effects of CUEDC2 on cerebral I/R-induced neuron insult.** (**A**, **B**) Apoptotic cell death in neurons as detected by flow cytometry with Annexin V/PI staining. (**C**) Apoptotic cell death in neurons as detected by LDH-leakage assay. (**D**) Neuron viability as assessed by the MTT assay. (**E**, **F**) Protein expression of caspase-3 in neurons as assayed by western blotting. (**G**) ROS production in neurons as detected by DCFH-DA assay. (**H**) MDA production in neurons as evaluated by lipid peroxidation MDA assay. (**I**) SOD production in neurons as determined by WST-8 assay. (**J**) T-AOC level in neurons as detected by ABTS assay. (**K**) Viability of H_2_O_2_-treated neurons as analyzed by MTT assay. (**L**) Apoptotic cell death in H_2_O_2_-treated neurons as detected by LDH leakage assay. CTR, control; CUEDC2: CUE domain-containing 2; OGD/R: oxygen-glucose deprivation (4 hours) and reperfusion (24 hours); neurons-siRNA-CUEDC2: small interfering RNA silencing CUEDC2 in neurons; neurons-vector: the vector of neurons. All data are presented as the mean value ± SD. *p<0.05, **p<0.01; compared to the vector group, the H_2_O_2_ treatment group.

Based on the above result, 4 hours OGD and 24 hours reperfusion time point was chose for the following experimental study. The expression of CUEDC2 was knocked down in neurons (Supplementary Figure 1). Compared to the control group, OGD/R insult significantly increased the rate of cell apoptosis and LDH leakage in neurons-vector group (Figure 2A–2C). CUEDC2 silencing in the neurons inhibited the apoptosis rate in the neurons-siRNA-CUEDC2 group when compared to the neurons-vector group. In addition, there was a reduction in LDH leakage in the neurons-siRNA-CUEDC2 group (Figure 2A–2C). Compared to the neurons-vector group, ablating CUEDC2 significantly enhanced the viability of the insulted neurons in the neurons-siRNA-CUEDC2 group (Figure 2D). Western blot revealed a marked suppression of caspase-3 expression in the neurons-siRNA-CUEDC2 group when compared to the neurons-vector group (Figure 2E–2F). These findings indicate that the decrease in CUEDC2 remarkably suppressed OGD/R-induced apoptotic cell death in neurons.

Excess oxidative stress responses are associated with apoptotic cell death in the neurons after cerebral I/R insult. The secretion of ROS and MDA were shown to be higher in the neurons-siRNA-CUEDC2 and the neurons-vector groups after OGD/R injury when compared to the control group (Figure 2G–2H). Conversely, the ROS and MDA levels were significantly low in the neurons-siRNA-CUEDC2 group compared to the neurons-vector group in the OGD/R-induced neuron insult. Furthermore, SOD and T-AOC levels were low in both neurons-siRNA-CUEDC2 and the neurons-vector groups following OGD/R challenge (compared to the control group). However, CUEDC2 silencing significantly upregulated SOD and T-AOC levels in the neurons-siRNA-CUEDC2 group compared to the neurons-vector group (Figure 2I–2J). When the neurons were co-treated with a pro-oxidant H_2_O_2_ following OGD/R insult, the MTT assay showed that the upregulated cell viability of neuron-siRNA-CUEDC2 group was markedly inhibited compared to the non-incubated neurons- siRNA-CUEDC2 group. The LDH leakage assay exhibited a significantly increase in neurons-siRNA-CUEDC2 group in combination with the H_2_O_2_ compared to the non-incubated neurons-siRNA-CUEDC2 group (Figure 2K–2L). These results imply that CUEDC2 exhibits its neuroprotective role in cerebral I/R by modulating oxidative stress levels in the neuron cells.

### MSCs attenuate cerebral I/R-induced injury in co-cultured neurons

**Figure 3:**
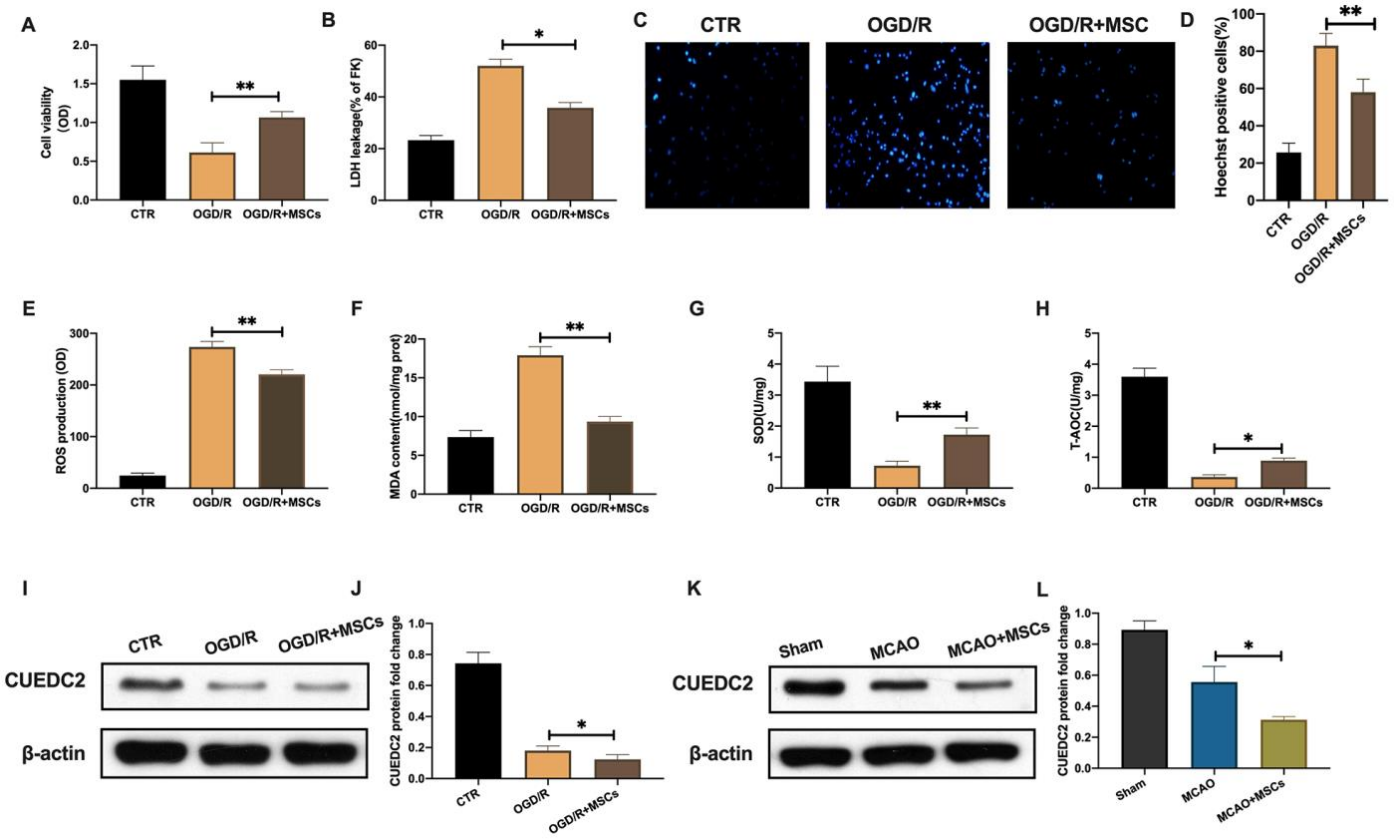
**MSCs attenuate cerebral I/R-induced injury in co-cultured neurons.** (**A**) Viability of co-cultured neurons as assessed by MTT assay. (**B**) Apoptotic cell death in co-cultured neurons as detected by LDH-leakage assay. (**C**, **D**) Apoptotic cell death in co-cultured neurons as detected by Hoechst staining. (**E**) ROS production in co-cultured neurons as detected by DCFH-DA assay. (**F**) MDA production in co-cultured neurons as evaluated by lipid peroxidation MDA assay. (**G**) SOD production in co-cultured neurons as determined by WST-8 assay. (**H**) T-AOC level in co-cultured neurons as detected by ABTS assay. (**I**, **J**) CUEDC2 protein expression in co-cultured neurons as assayed by western blotting. (**K**, **L**) CUEDC2 protein expression in MSCs-treated brain tissues as assayed by western blotting. CTR, control; CUEDC2: CUE domain-containing 2; OGD/R: oxygen-glucose deprivation (4 hours) and reperfusion (24 hours); MSCs: mesenchymal stem cells; MCAO, middle cerebral artery occlusion. All data are presented as the mean value ± SD. *p<0.05, **p<0.01; compared to OGD/R + MSCs group, MCAO + MSCs treatment group.

OGD/R significantly decreased cell viability in neurons. While, MSCs co-cultured with neurons showed a significantly upregulation of cell viability in the insulted neurons induced by OGD/R(Figure 3A). In addition, LDH leakage assays showed that neurons co-cultured with MSCs exhibited a remarkably inhibited OGD/R associated cell death (Figure 3B). These findings are concurrent with those of the Hoechst staining assay (Figure 3C–3D). Regarding oxidative stress in the co-cultured neurons, ROS and MDA levels were remarkably elevated in the injured neurons after OGD/R insult. However, these levels were suppressed upon co-culture with MSCs (Figure 3E–3F). In addition, the reduction of SOD and T-AOC levels were significantly rescued in co-cultured neurons after MSCs treatment under OGD/R environment (Figure 3G–3H). The above results indicate that MSCs can attenuate cerebral I/R-induced insult in co-cultured neurons.

### MSCs promote CUEDC2 degradation in the brain upon cerebral I/R insult

Compared to the control group there was significantly low protein levels of CUEDC2 in neurons upon OGD/R insult. Moreover, MSCs treatment further inhibited CUEDC2 expression in co-cultured neurons after OGD/R challenge (Figure 3I–3J). Compared to the sham group, cerebral I/R insult significantly suppressed CUEDC2 protein expression in brain tissues. Further, the injection with MSCs group promoted the downregulation of CUEDC2 expression following cerebral I/R challenge (Figure 3K–3L). These results imply that MSCs treatment enhanced the degradation of CUEDC2 in the brain tissues as well as in the neuronal cells after cerebral I/R insult. Therefore, CUEDC2 might be a potential target for MSCs when protecting against cerebral I/R induced neuronal injury.

### CUEDC2 knockdown in MSCs suppresses OGD/R-induced apoptosis in co-cultured neurons

**Figure 4:**
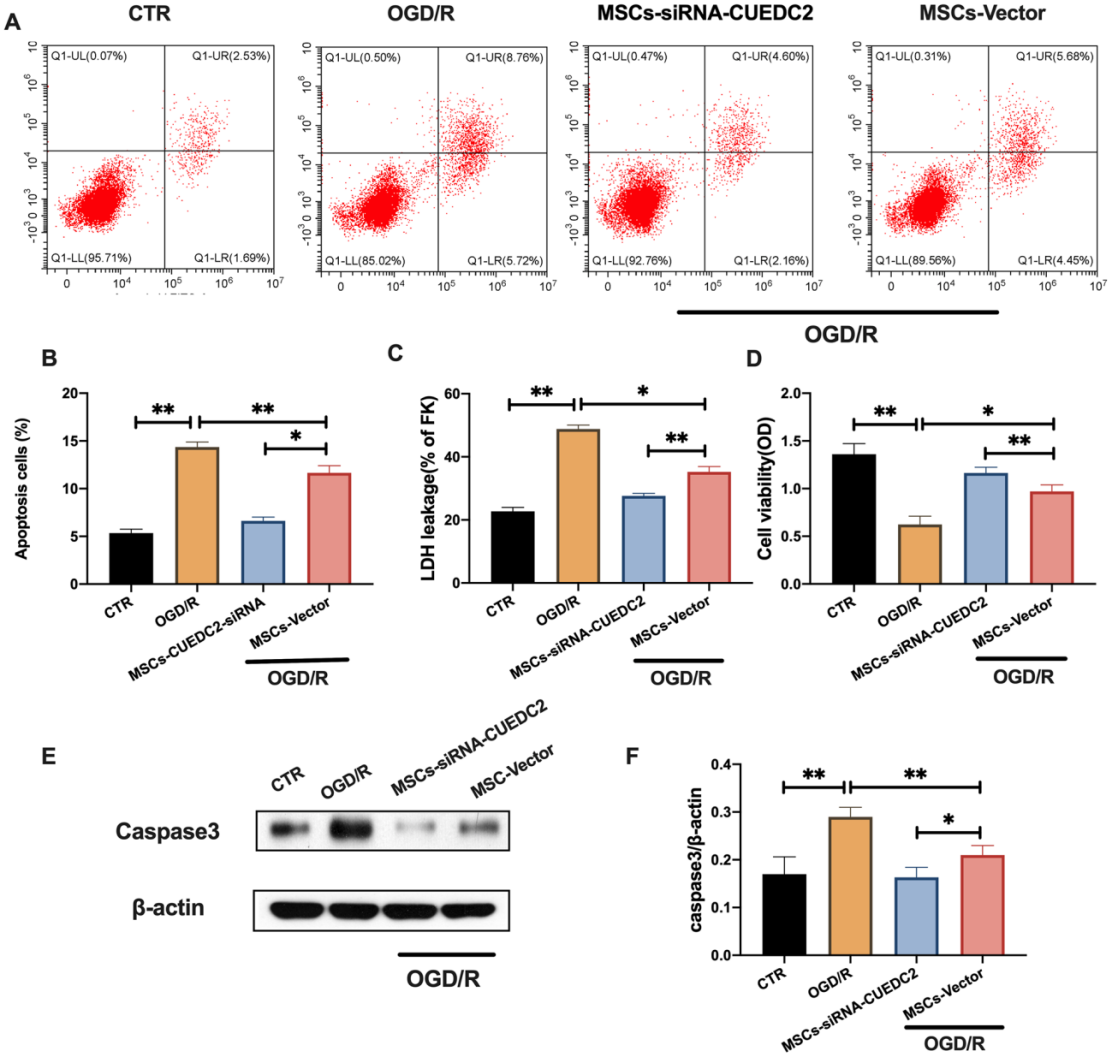
**CUEDC2 knockdown in MSCs enhances the inhibitory effect on OGD/R-induced apoptotic cell death in co-cultured neurons.** (**A**, **B**) Apoptotic cell death in co-cultured neurons after treatment with different MSCs as detected by flow cytometry with Annexin V/PI staining. (**C**) Apoptotic cell death in co-cultured neurons after treatment with different MSCs as analyzed by LDH-leakage assay. (**D**) Viability of co-cultured neurons after treatment with different MSCs as evaluated by MTT assay. (**E**, **F**) Caspase-3 protein expression in co-cultured neurons after treatment with different MSCs as assayed by western blotting. CTR: control; MSCs: mesenchymal stem cells; CUEDC2: CUE domain-containing 2; OGD/R: oxygen-glucose deprivation (4 hours) and reperfusion (24 hours); MSCs- siRNA-CUEDC2: small interfering RNA silencing CUEDC2 in MSCs; MSCs-vector: the vector of MSCs. All data are presented as the mean value ± SD. *p<0.05, **p<0.01; compared to the control group, MSCs-vector group.

CUEDC2 knockdown in MSCs was performed (Supplementary Figure 2) to determine whether CUEDC2-modified MSCs could enhance the neuroprotective effects on the insulted neurons. There was a significantly low apoptotic rate in co-cultured neurons in the MSCs-siRNA-CUEDC2 group compared to the MSCs-vector group after OGD/R insult (Figure 4A–4B). These findings were consistent with the results of LDH leakage alteration during OGD/R injury in co-cultured neurons with MSCs-siRNA-CUEDC2 treatment (Figure 4C). MTT assays showed that cell viabilities in the MSCs-siRNA-CUEDC2 group were significantly enhanced after co-culture compared to the MSCs-vector group (Figure 4D). Moreover, compared to MSCs-vector group, MSCs-siRNA-CUEDC2 group exhibited significantly decreased caspase-3 expression levels after OGD/R neuron insult (Figure 4E–4F). Taken together, these findings imply that CUEDC2 knockdown in MSCs enhanced anti-apoptotic effect on OGD/R-induced neuronal impairment.

### CUEDC2-degraded MSCs protect against OGD/R-induced apoptotic cell death in co-cultured neurons by suppressing oxidative toxicity

**Figure 5:**
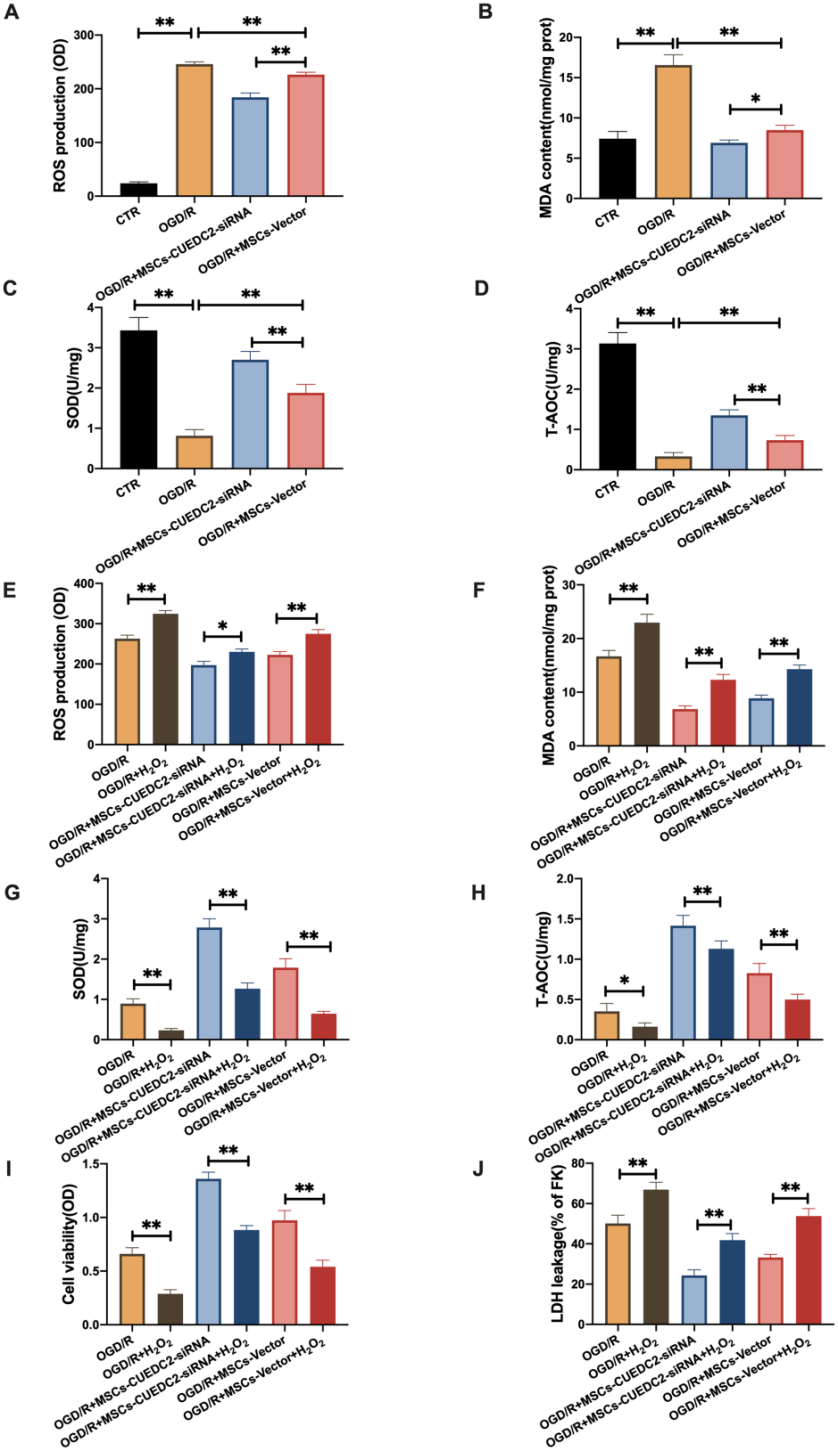
**CUEDC2 degradation in MSCs improves protection against OGD/R-induced apoptotic cell death in co-cultured neurons by suppressing oxidative toxicity.** (**A**) ROS production in co-cultured neurons after treatment with different MSCs as detected by DCFH-DA assay. (**B**) MDA production in co-cultured neurons after treatment with different MSCs as evaluated by lipid peroxidation MDA assay. (**C**) SOD production in co-cultured neurons after treatment with different MSCs as determined by WST-8 assay. (**D**) T-AOC level in co-cultured neurons after treatment with different MSCs as detected by ABTS assay. (**E**) ROS production in co-cultured neurons after treatment with different MSCs exposed to H_2_O_2_ as analyzed by DCFH-DA assay. (**F**) MDA production in co-cultured neurons after treatment with different MSCs exposed to H_2_O_2_ as evaluated by lipid peroxidation MDA assay. (**G**) SOD production in co-cultured neurons after treatment with different MSCs exposed to H_2_O_2_ as determined by WST-8 assay. (**H**) T-AOC level in co-cultured neurons after treatment with different MSCs exposed to H_2_O_2_ as detected by ABTS assay. (**I**) Co-cultured neuron viability after treatment with different MSCs subjected to H_2_O_2_ as evaluated by MTT analysis. (**J**) Apoptosis in co-cultured neurons after treatment with different MSCs subjected to H_2_O_2_ as evaluated by LDH leakage assay. CTR: control; MSCs: mesenchymal stem cells; CUEDC2: CUE domain-containing 2; OGD/R: oxygen-glucose deprivation (4 hours) and reperfusion (24 hours). MSCs- siRNA-CUEDC2: small interfering RNA silencing CUEDC2 in MSCs; MSCs-vector: the vector of MSCs. All data are presented as the mean value ± SD. *P<0.05, **P<0.01; compared with the control, MSCs-vector, and H_2_O_2_ treatment groups.

Previous studies have documented that CUEDC2 inhibits ROS production and regulates redox-associated signaling pathways in cardiomyocytes after I/R stimulation [21]. We have also shown that oxidative stress responses to OGD/R stimulation were suppressed by the downregulated CUEDC2 levels in neurons. Thus, we determined whether CUEDC2 silencing in MSCs could enhance the antioxidative stress effect of MSCs on the injured neurons. The results showed significantly low neuronal ROS and MDA levels in the MSCs-siRNA-CUEDC2 group compared to the MSCs-vector group after OGD/R challenge (Figure 5A–5B). Furthermore, CUEDC2 knockdown in MSCs enhanced the antioxidant effect by significantly elevating SOD and T-AOC levels in the co-cultured neurons compared to the MSCs-vector group (Figure 5C–5D).

To confirm this effect, we eliminated CUEDC2 activity by pro-oxidant H_2_O_2_ treatment in MSCs. The results showed that ROS and MDA levels were significantly elevated after H_2_O_2_ pretreatment when MSCs-siRNA-CUEDC2 were co-cultured with neurons compared to the non-treated MSCs-siRNA-CUEDC2 group (Figure 5E–5F). However, there was a marked reduction of SOD and T-AOC after H_2_O_2_ induced CUEDC2 knockdown in the MSCs group (Figure 5G, 5H). Collectively, these results imply that siRNA-CUEDC2 in MSCs might play a critical role in enhancing antioxidant effects in the neurons under OGD/R conditions.

Moreover, we evaluated whether the enhanced protection against OGD/R-induced apoptosis in co-cultured neurons was regulated by the antioxidant effect of CUEDC2-modified MSCs. Under OGD/R conditions, cell viability was higher in the group co-cultured with MSCs-siRNA-CUEDC2. However, H_2_O_2_ pretreatment in this group led to a decrease in cell viability (Figure 5I). It was also found that siRNA-CUEDC2 significantly reduced LDH leakage in MSCs co-cultured with neurons following OGD/R insult (Figure 5J). In contrast, H_2_O_2_ treatment enhanced LDH leakage in the group co-cultured with MSCs-siRNA-CUEDC2 following OGD/R injury compared to the non-treated group (Figure 5J). Taken together, these results confirmed that CUEDC2 generates an enhanced antioxidant effect that enables MSCs to prevent apoptotic cell death caused by OGD/R.

### CUEDC2 ablation enhances the efficacy of MSCs in I/R-induced brain damage

**Figure 6:**
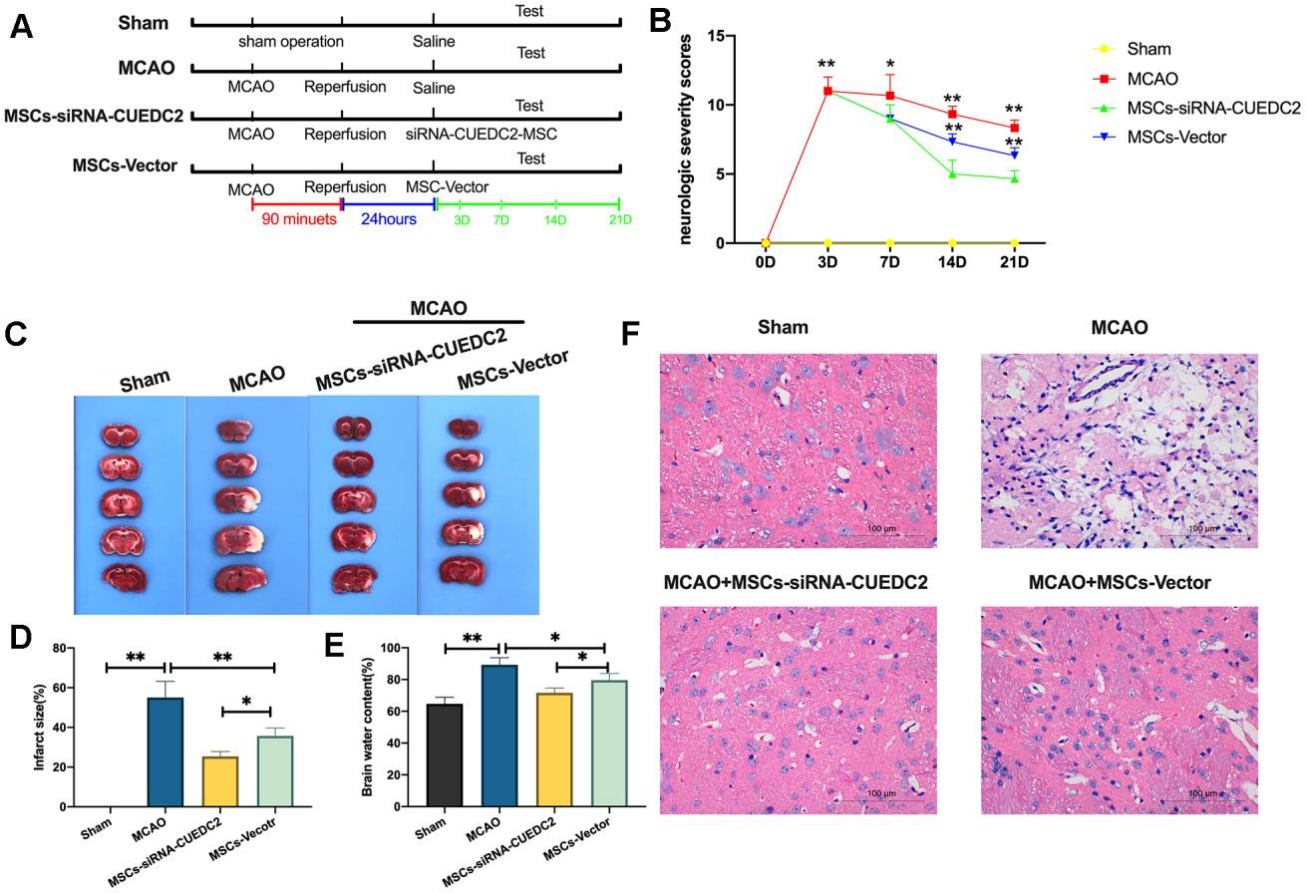
**CUEDC2 ablation promotes the efficacy of MSCs in I/R-induced brain damages.** (**A**) Illustration of MCAO-induced model of cerebral I/R in rats and different MSCs treatment. SD rats were exposed to MCAO for 90 minutes and subsequent reperfusion. After 24-hours of reperfusion, the CUEDC2-modified MSCs and the MSCs vector were infused on MCAO model rats. (**B**) Evaluation of neurologic severity scores. (**C**, **D**) Assessment of infarct size with TTC staining in whole-brain tissues. (**E**) Determination of brain water content. (**F**) H&E staining in brain tissues. Scale bar = 100 μm. MSCs: mesenchymal stem cells; MSCs- siRNA-CUEDC2: small interfering RNA silencing CUEDC2 in MSCs; MSCs-vector: the vector of MSCs; MCAO, middle cerebral artery occlusion. All data are presented as the mean value ± SD. *p<0.05, **p<0.01; compared to sham group, the vector of MSC group.

We further evaluated whether CUEDC2 ablation would enhance the effectiveness of MSCs in I/R-induced brain damage in rats (Figure 6A). It was found that, compared to the sham group, the MCAO group had significantly elevated neurologic severity scores from the third day after operation (Figure 6B). However, at 7, 14 and 21 days, the administration of MSCs markedly lowered the neurologic deficit score after MCAO operation in MSCs-siRNA-CUEDC2+MCAO and MSCs-vector+MCAO treatment groups. In addition, CUEDC2 ablation enhanced the effect of MSCs on neurologic deficit scores at 14- or 21-days post MCAO procedure compared to the MSCs-vector+MCAO treatment group. Thus, we assayed the brain infarct size using TTC staining at 14 days post MCAO operation. The results revealed a marked infarct in the brain caused by I/R insult in the MCAO group. Conversely, the results showed a significantly smaller infarct size in the MSCs-vector+MCAO group and the MSCs-siRNA-CUEDC2 +MCAO group compared to the MCAO group (Figure 6C–6D). Moreover, compared to the MSCs-Vector + MCAO group, CUEDC2 knockdown in MSCs (MSCs-siRNA-CUEDC2 +MCAO group) effectively suppressed the MCAO injury induced infarct size (Figure 6C–6D). Next, the brain water content was determined 14 days after MCAO operation. It was found that the brain water content was markedly high in the MCAO model compared to the sham group. However, the brain water content was reduced by MSCs injection in the MSCs-siRNA-CUEDC2+MCAO group and the MSCs-vector +MCAO group. Furthermore, compared to the MSCs-vector+MCAO group, CUEDC2 silencing in MSCs (MSCs-siRNA-CUEDC2 +MCAO group) was found to significantly enhance the effect of MSCs in reducing the brain water content in the MCAO model (Figure 6E). Additionally, consistent findings were obtained by H&E staining (Figure 6F). Taken together, these results indicated that CUEDC2 knockdown enhanced the protective effects of MSCs on ameliorating cerebral I/R-induced brain damage.

### CUEDC2 knockdown in MSCs upregulates antioxidative effects on the co-cultured neurons by elevating GPX1 expression

**Figure 7:**
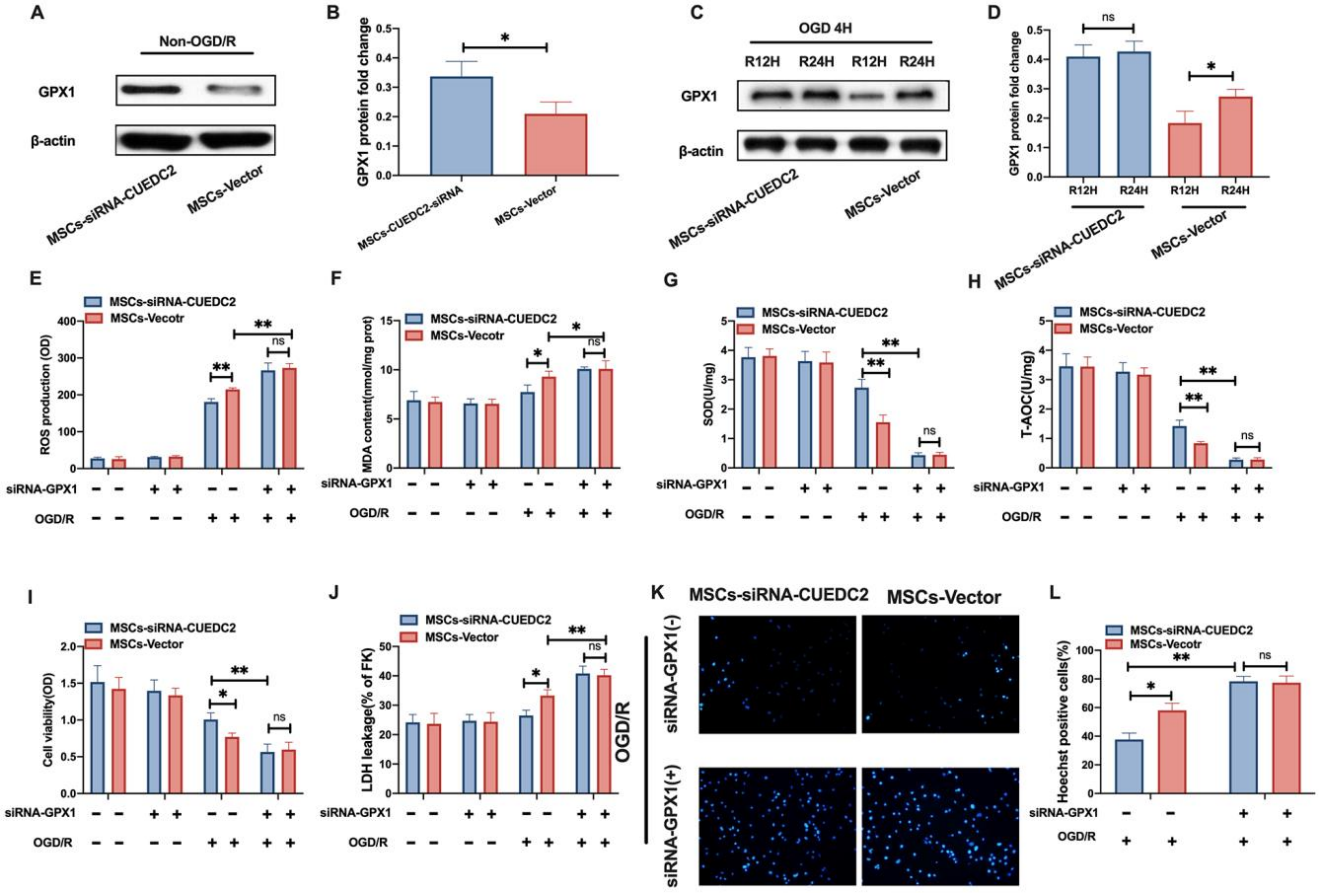
**CUEDC2 silencing in MSCs upregulates the antioxidant effect on co-cultured neurons by increasing GPX1 expression**. (**A**, **B**) GPX1 protein expression in co-cultured neurons under non-OGD/R condition as detected by western blotting. (**C**, **D**) GPX1 protein expression in co-cultured neurons under OGD/R condition as detected by western blotting. (**E**) ROS production in co-cultured neurons after treatment with various MSCs as analyzed by DCFH-DA assay. (**F**) MDA production in co-cultured neurons after treatment with various MSCs as evaluated by lipid peroxidation MDA assay. (**G**) SOD production in co-cultured neurons after treatment with various MSCs as determined by WST-8 assay. (**H**) T-AOC level in co-cultured neurons after treatment with various MSCs as detected by ABTS assay. (**I**) Viability of co-cultured neurons after treatment with various MSCs as evaluated by MTT analysis. (**J**) Apoptosis in co-cultured neurons after treatment with various MSCs as evaluated by LDH leakage assay. (**K**, **L**) Apoptosis in co-cultured neurons after treatment with various MSCs as detected by Hoechst staining. CUEDC2: CUE domain-containing 2; OGD/R: oxygen-glucose deprivation (4 hours) and reperfusion (12- or 24-hours). MSCs-siRNA-CUEDC2: small interfering RNA silencing CUEDC2 in MSCs; MSCs-vector: the vector of MSCs; siRNA-GPX1: small interfering RNA knockdown of GPX1 in MSC; All data are presented as the mean value ± SD. *p<0.05, **p<0.01; comparison to MSC-vector group.

Several studies have documented that the antioxidant effect of GPX1 is involved in preventing cerebral I/R-induced insult [22]. Jian et al. reported that CUEDC2 regulates oxidative stress capacity in cardiomyocytes by maintaining GPX1 stability [21]. Thus, we investigated the potential mechanism by which CUEDC2-modified MSCs protected against cerebral I/R-induced injury *in vitro*. We focused on GPX1 expression in neurons co-cultured with the MSCs vector and ablation of CUEDC2 in MSCs in non-OGD/R and OGD/R conditions, respectively. The results revealed that the protein expression levels of GPX1 were significantly higher in neurons co-cultured with the MSCs-siRNA-CUEDC2 group compared to the MSCs-vector group under non-OGD/R simulation (Figure 7A–7B). Moreover, under OGD/R conditions, neurons co-cultured with the MSCs-vector group exhibited a marked upregulation of GPX1 protein expression at 24 hours reperfusion time point compared to 12 hours reperfusion time point after 4-hours of OGD insult (Figure 7C–7D). CUEDC2 silencing in MSCs led to a sustained increase in the GPX1 levels in co-cultured neurons at either 12- or 24-hours of OGD/R (Figure 7C–7D). Next, the expression of GPX1 was then silenced in MSCs (Supplementary Figure 3). The results showed that there were significantly high ROS production levels in neurons co-cultured with only siRNA-GPX1 in the MSCs group (MSCs-siRNA-GPX1) compared to the non-GPX1-silenced MSCs group (MSCs-vector) after OGD/R challenge. Interestingly, when both GPX1 and CUEDC2 were knocked down in MSCs (MSCs-siRNA-GPX1/CUEDC2), the modified MSCs lost their antioxidative role, including the upregulated ROS and MDA production, as well as downregulated SOD and T-AOC levels observed in co-cultured neurons following OGD/R insult compared to the only siRNA-GPX1 MSCs group (Figure 7E–7H). Meanwhile, cell viability and LDH leakage analysis revealed that there was no significant difference between in silenced GPX1 expression in the siRNA-CUEDC2 group(MSCs-siRNA-GPX1/CUEDC2) and the only siRNA-GPX1 MSCs group (MSCs-siRNA-GPX1) (Figure 7I–7J). In GPX1 ablation, similar findings were shown by the Hoechst assay in the corresponding group (Figure 7K–7L). Collectively, these findings indicate that GPX1 might be a critical downstream effector of CUEDC2 that is involved in the neuroprotective role of MSCs under the cerebral I/R environment.

### CUEDC2 knockdown in MSCs decreases NF-κB activation following cerebral I/R injury

**Figure 8:**
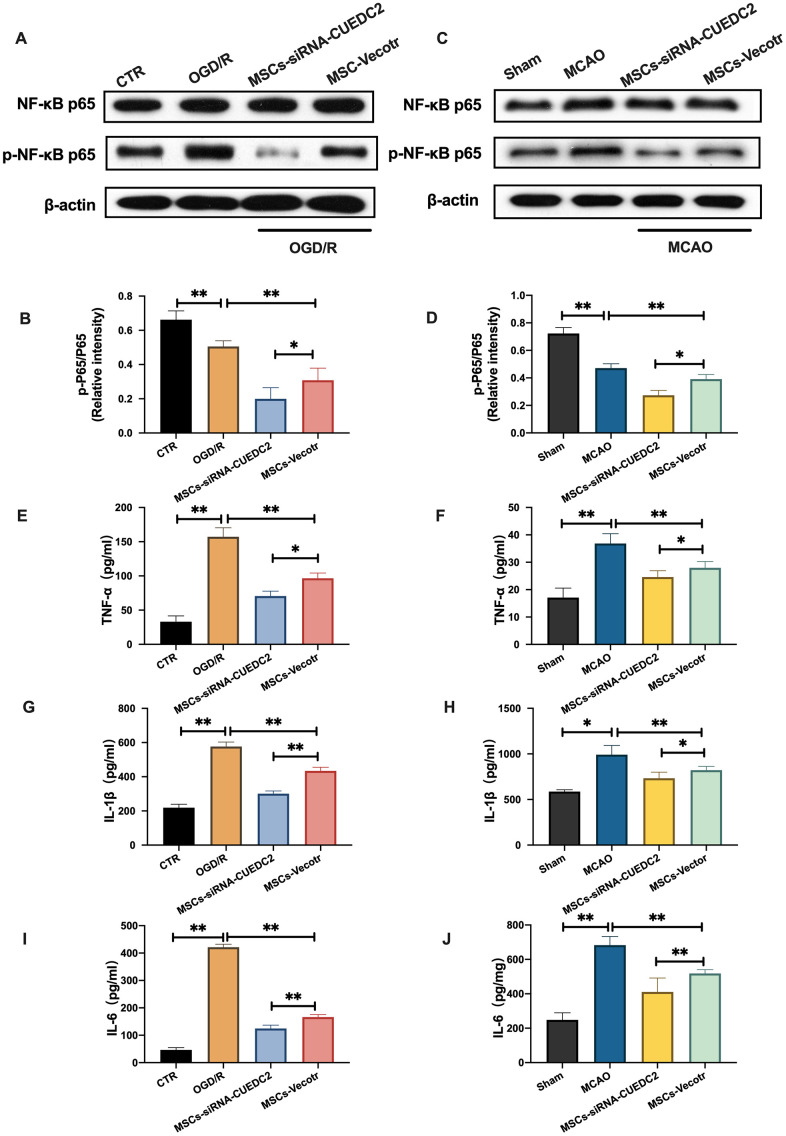
**CUEDC2 silencing in MSCs suppresses NF-kB activity following cerebral I/R injury.** (**A**, **B**) NF-kB and p-NF-kB protein expression in co-cultured neurons as detected by western blotting. (**C**, **D**) NF-kB and p-NF-kB protein expression in brain tissues as detected by western blotting. (**E**) TNF-α level in co-cultured neurons as detected by ELISA assay. (**F**) TNF-α level in brain tissues as analyzed by ELISA assay. (**G**) IL-1β level in co-cultured neurons as determined by ELISA assay. (**H**) IL-1β level in brain tissues as analyzed by ELISA assay. (**I**) IL-6 level in co-cultured neurons as evaluated by ELISA assay. (**J**) IL-6 level in brain tissues as determined by ELISA assay. CTR: control; CUEDC2: CUE domain-containing 2; OGD/R: oxygen-glucose deprivation (4 hours) and reperfusion (24 hours). MSCs: mesenchymal stem cells; MSCs-siRNA-CUEDC2: small interfering RNA silencing CUEDC2 in MSCs; MSCs-vector: the vector of MSCs; MCAO, middle cerebral artery occlusion. All data are presented as the mean value ± SD. *p<0.05, **p<0.01; compared to the control, sham and the vector of MSC-group.

Numerous studies have confirmed that NF-kB activation plays a significant role in cerebral I/R [23, 24]. We analyzed the activation of NF-kB in brain tissues and neurons under cerebral I/R condition. The results from the western blot assay revealed a significant increase in NF-kB p65 phosphorylation after cerebral I/R insult *in vivo* and *in vitro* compared to the sham group in brain tissues and the control groups in co-cultured neurons, respectively. However, compared to the injured neurons in the OGD/R group, the neurons co-cultured with the MSCs-vector group inhibited NF-kB p65 phosphorylation upon OGD/R insult (Figure 8A–8B). Moreover, MSCs-vector injection significantly reduced the expression of NF-kB p65 phosphorylation in the MSCs-Vector + MCAO group relative to the MCAO group (Figure 8C–8D). CUEDC2 knockdown (MSCs-siRNA-CUEDC2 group) enhanced the neuroprotective effect of MSCs on suppression of the elevated NF-kB p65 phosphorylation in co-cultured neurons compared to the non-vector group (MSCs-vector group) after OGD/R-induced injury (Figure 8A–8B). Consistent with the *in vitro* results, a remarkable suppression of NF-kB p65 phosphorylation was exhibited in the MSCs-siRNA-CUEDC2 + MCAO group compared to the MSCs-Vector + MCAO group (Figure 8C–8D). Furthermore, compared to the control and sham groups, TNF-α, IL-1β and IL-6 levels in neurons and brain tissues were elevated under cerebral I/R stimulation. However, CUEDC2 knockdown (MSCs-siRNA-CUEDC2+OGD/R group and MSCs-siRNA-CUEDC2+MCAO group) enhanced the protective effect of MSCs by inhibiting the production of TNF-α, IL-1β and IL-6 in co-cultured neurons and brain tissues during cerebral I/R challenge compared to MSCs-vector +OGD/R group and MSCs-Vector + MCAO group, respectively (Figure 8E–8J). Based on these results, the elevated expression levels of NF-kB under the cerebral I/R environment could be suppressed by CUEDC2 knockdown in MSCs. Taken together, these findings imply that CUEDC2 silencing in MSCs might play a functional role in modulating the activation of NF-kB to ameliorate inflammatory responses upon cerebral I/R-induced insult.

## DISCUSSION

Studies indicate that MSCs may be a promising approach for managing cerebral I/R-induced injury [25, 26]. However, the repair and restoration of injured brain tissues or neurons is challenging, and the effects of MSCs in this processes has not been established. The existing data from clinical trials are not sufficient to draw definitive conclusions [9]. In addition, the low survival rate of engrafted MSCs due to the harsh microenvironment of cerebral I/R cannot be ignored [7, 8]. Therefore, it is necessary to identify novel approaches to improve the therapeutic efficacy of transplanted MSCs on the impaired neurons.

Previous studies have shown that CUEDC2 is a regulator for progesterone and estrogen receptors in breast cancer [13, 27]. It is also a critical tumor suppressor for different types of cancers because it is involved in the regulation of inflammatory responses and cell proliferation [15, 28]. Kim et al. have reported that CUEDC2 modulates bone formation and osteoblast differentiation by activating the SOCS3-STAT3 signaling pathway [20]. However, the functional role of CUEDC2 in cerebral I/R has not been established. Jian et al found that CUEDC2 expression in the heart elevated the antioxidant capacity in cardiomyocytes and ROS scavenging capacity by modulating GPX1 stability during cardiac I/R [21]. In this study, there was a suppressed CUEDC2 protein expression in neurons and brain tissues after cerebral I/R challenge. However, there was no noticeable alterations in the mRNA expressions of CUEDC2. The lack of changes in mRNA expression levels could have contributed to the regulation of CUEDC2 and might be regulated by posttranscriptional modification. Moreover, compared to the neurons-vector group, CUEDC2 silencing significantly inhibited apoptotic cell death and oxidative stress in neurons following *in vitro* cerebral I/R. When neurons-siRNA-CUEDC2 were pretreated with pro-oxidant H_2_O_2_, the increased cell viability and decreased cell death rate were suppressed and upregulated, respectively, during OGD/R insult. These findings were consistent with CUEDC2 expression under the heart I/R condition and in glioma and glioma cells [18, 21]. Therefore, CUEDC2 might serve as a novel neuroprotective target that is involved in the modulation of apoptosis through a cerebral I/R-induced intrinsic antioxidant defense mechanism.

Several feasible strategies have been proposed to enhance the therapeutic efficacy of MSCs in managing cerebral I/R-induced insult. The oxidative stress environment of cerebral I/R is severe for MSCs survival, eventually affecting their therapeutic outcomes [29, 30]. Therefore, promotion of the antioxidant capacity of MSCs in unfavorable conditions could be a potential target for enhancing their therapeutic effects. In this study, MSCs were shown to partially rescue apoptotic cell death and oxidative stress responses in damaged neurons. Of mention, it seems that there was no obviously visible difference both in OGD/R group and OGD/R+MSC group (Figure 3I). But after statistical analysis, there was a significantly difference(p=0.0282) of the protein expression of CUEDC2 between OGD/R group and OGD/R+MSC group. Moreover, the similar data can be noted in the in *vivo* experiment. We suggested that the inhibition of CUEDC2 expression was attributed to the protective ability of MSCs in co-cultured neurons. CUEDC2, therefore, might be a potential target for enhancing the therapeutic efficacy of MSCs in response to cerebral I/R.

Nakajima et al. documented that upregulated interleukin-10(IL-10) in MSCs enhanced neuroprotection in acute ischemic stroke-induced brain injury in the MCAO model [12]. Consistent with this report, CUEDC2-modified MSCs treatment (MSCs -siRNA-CUEDC2 group) significantly reduced the infarct size, brain water content and apoptosis in brain tissues and enhanced neurologic behaviors in the MCAO model compared to the MSCs-Vector +MCAO group. These findings were similar to the protective role of CUEDC2 in the neurons and the heart under the I/R environment as previously reported. Conclusively, CUEDC2 knockdown enhanced the therapeutic efficacy of MSCs in cerebral I/R.

Moreover, CUEDC2 ablation in MSCs (MSCs -siRNA-CUEDC2 group) promoted antioxidant effects that protect against cerebral I/R-induced apoptotic cell death in neurons and brain impairment. GPX1 plays a significant neuroprotective role in various neurological disorders, such as Parkinson’s disease [31] and cerebral ischemic stroke [32], through its antioxidant function. GPX1 expression is enriched in the brain tissues. Haan et al. found that GPX1 knockdown resulted in a chronic pro-oxidant environment in mice [33] while Wong et al found that GPX1 silencing accelerated cerebral ischemia-induced impairments through upregulated ROS generation and damaged vascular permeability [34]. More importantly, Zhan et al reported that the antioxidant effect of CUEDC2 in cardiomyocytes was modulated by GPX1 [21]. In this study, GPX1 expression was higher in the MSCs -siRNA-CUEDC2 group co-cultured with neurons compared to the MSCs-vector group in either non-OGD/R or OGD/R condition. These findings suggest that the enhanced therapeutic efficacy in MSCs -siRNA-CUEDC2 might be associated with the elevated expression of GPX1. Consistent with previous studies [21], our findings implied that upregulated GPX1 expression might exert a neuroprotective role in cerebral ischemic stroke. The absence of GPX1 in MSCs exacerbated cerebral I/R-induced neuron injury, including an enhanced level of oxidative stress and increased apoptotic cell death. Interestingly, there were no significant differences between MSCs-siRNA-GPX1/CUEDC2 group and only MSCs-siRNA-GPX1 group. When GPX1 and CUEDC2 were absent in MSCs, the modified MSCs exhibited a suppressed therapeutic effect in response to the cerebral I/R environment. These findings imply that GPX1 is a critical protective downstream target that enhanced the therapeutic efficacy of CUEDC2-modified MSCs in cerebral I/R-induced neuron impairment.

Studies have confirmed that CUEDC2 is involved in inflammatory responses induced by different types of cancers [28, 35]. There was a remarkably low activation of NF-kB in neurons co-cultured with MSCs -siRNA-CUEDC2 as well as in MSCs-siRNA-CUEDC2 injection brain tissues when compared to the MSCs-vector group under cerebral I/R condition. This was accompanied by suppressed levels of pro-inflammatory indices, including TNF-α, IL-1β and IL-6, in the CUEDC2-modified MSCs group (MSCs-siRNA-CUEDC2 group) than MSCs-vector group after cerebral I/R insult *in vitro* and *in vivo*. These results were consistent with those of Li et al. [18], who found that CUEDC2 regulated IkB kinase (IKK) and protein phosphatase 1 (PP1) expression, eventually repressing the activation of NF-kB to influence inflammatory outcomes in the cell cycle. In contrast, Zhang et al documented that there is a negative modulator between CUEDC2 and NF-kB pathway in chronic myeloid leukemic cells [19]. Based on these contrasting findings, we concluded that the role of CUEDC2 in regulating the activation of NF-kB is determined by the type of tissue, cell and system. Therefore, our findings confirmed that the NF-kB pathway is involved in enhancing the therapeutic efficacy of CUEDC2-modified MSCs on impaired neurons under cerebral I/R challenge.

## CONCLUSIONS

In conclusion, we present a novel neuroprotective role of CUEDC2 in cerebral I/R insult. CUEDC2-modified MSCs exert their therapeutic efficacy on insulted neurons in cerebral I/R by activating the NF-kB signaling pathway. In addition, GPX1 is a critical protective target that enhances the therapeutic effect of CUEDC2 in MSCs. This study provides novel insights into the use of MSCs as a therapeutic strategy for enhancing the clinical outcomes of cerebral ischemic stroke.

## MATERIALS AND METHODS

All the experimental methods in this study were approved by the Ethical Committee of the Hunan Normal University(NO.2020203).

### Culturing of MSCs and primary neurons

MSCs were purchased from Procell Biotech (Wuhan, China). They were inoculated in Dulbecco’s modified Eagle’s medium (DMEM, Invitrogen, USA) comprising 10% fetal bovine serum (FBS, Invitrogen, USA) and supplemented with 100 U/ml penicillin and 100 μg/ml streptomycin, and incubated at 37° C and 5% CO_2_.

As previously described [36], primary neurons were obtained from Sprague-Dawley (SD) rat embryos on the 16 th day of gestation. The dissected brain tissues were digested using papain at 37° C for 10 minutes. The cells were plated on 18-mm glass dishes containing poly-D-lysine (Sigma, USA) at an approximate density of 200,000 cells/well in a 12-well plate with cultured medium (DMEM combined with 10% FBS). After 24 hours, the cultured medium was replaced with neurobasal medium supplemented with B27 (Invitrogen, USA) as well as Glutamax (Invitrogen, USA) and incubated at 37° C with 5% CO_2_.

### Oxygen-glucose deprivation and reperfusion model

To mimic the cerebral I/R microenvironment, an oxygen-glucose deprivation and reperfusion (OGD/R) model was utilized *in vitro*. Briefly, the cells were incubated in glucose-free Hanks’ balanced salt solution (D-Hank’s, Biological Industries, Northern Kibbutz Beit Haemek, Israel) in an incubator (5% CO_2_, 95% N_2_ and 37° C) for 4 hours to simulate an ischemic environment. Thereafter, they were restored to traditional cell incubator conditions of 37° C and 5% CO_2_ atmosphere. The D-Hank’s medium was replaced with the respective MSCs and primary neurons medium, and then cultured at different time points to simulate the reperfusion process according to the experimental requirement.

### Co-culture treatment system

The MSCs were co-cultured with the primary neurons in 6-well transwell plates (Corning, USA) in the upper and lower chambers, respectively [11, 37]. After 24 hours of co-culture, the cells were evaluated using the following experiments.

### Cell viability and apoptosis assays

After the OGD/R-induced injury, cell viability was evaluated using the MTT assay kit (Beyond, Shanghai, China) as described by the manufacturer’s instructions. A microplate reader (Thermo Fisher, USA) was used to measure absorbance at 450 nm. The apoptotic rate was determined by flow cytometry using Annexin-V/PI apoptosis kit (Beyond, Shanghai, China) as previously described [38]. Fluorescence was recorded by flow cytometry (FACSCalibur, Becton-Dickinson, Sunnyvale, CA). The integrity of the plasma membrane is associated with cell apoptosis. Therefore, cell apoptosis was also determined by the level of lactate dehydrogenase (LDH) leakage from the damaged cells. The supernatants were obtained from the cells and operated under the manufacturer’s instruction of the LDH assay detection kit (Beyond, Shanghai, China).

### Hoechst staining assay

After OGD/R-induced injury, cell apoptosis was determined by Hoechst staining. Briefly, injured neuron cells were fixed in 4% PFA for 30 minutes at room temperature, and then washed twice using PBS. Thereafter, cells were incubated with Hoechst staining 33258 (Beyond, Shanghai, China) for 5 minutes at room temperature. The Hoechst staining medium was finally removed by washing twice using PBS, followed by observation under a fluorescence microscope (Olympus, Tokyo, Japan).

### Western blot analysis

Protein expression was analyzed by western blot following a previously described protocol [39]. Briefly, proteins were extracted from the cells and separated using SDS-PAGE. The proteins were transferred to PVDF membranes, which were then blocked and incubated with primary and secondary antibodies, respectively. These antibodies are presented in Supplementary Table 1. Blot images were generated using the ECL reagent kit (Advansta, Can, USA).

### Real-time PCR

Total RNA from the cell lysates were isolated using RNAprep Pure Cell kit (Tiangen, Beijing, China) according to the manufacturer’s instructions. Quantitative real-time PCR (qRT-PCR) was performed using SYBR Green Master Mix (Qiagen, Shanghai, China). The expression levels of CUECD2 and GPX1 were determined by a 2-ΔΔCt calculation method.

### Cell transfection

To silence CUEDC2 and GPX1 expression in MSCs, respectively, small interfering RNAs (siRNAs) targeting CUEDC2 and GPX1 were purchased from the GeneChem Biotech (Shanghai, China). The siRNA-CUEDC2 and siRNA-GPX1 target sequences were applicated, as well as their negative control plasmids, were then transfected into MSCs using Lipofectamine 2000, respectively (Invitrogen, Carlsbad, CA, United States) according to the manufacturer’s guidelines. The transfection efficiency was evaluated by western blot and PCR assays. The MSCs were then allocated into the following groups for the *in vitro* experimental assays: i. control group; ii. OGD/R group; iii. neurons-siRNA-CUEDC2 group (for neurons); iv. neurons-vector group (for neurons); v. MSCs-siRNA-CUEDC2 group (for MSCs) and vi. MSCs-vector group (for MSCs).

### Middle cerebral artery occlusion model and MSCs engraftation

Twenty-four male SD rats were purchased from Wellbio Biotech (Wellbio, Changsha, China) and used to evaluate the therapeutic efficacy of gene-modified MSCs and non-genetic MSCs on cerebral I/R insult *in vivo* through a classical middle cerebral artery occlusion (MCAO) model in rats. The SD rats were randomly distributed into the following groups for the *in vivo* experimental evaluations: i. sham group; ii. MCAO group; iii. MSCs-siRNA-CUEDC2 + MCAO group; and iv. MSCs-Vector + MCAO group. Every group contained six SD rats. The MCAO treatment procedures were performed as previously described. Briefly, the SD rats were anesthetized using 2.0%-3.0% isoflurane and maintained with 1.0%-1.5% isoflurane (containing in 70%N_2_O/30%O_2_) [40]. Subsequently, the right common carotid artery (CCA), internal carotid artery (ICA), and external carotid artery (ECA) were exposed using ophthalmic scissors. A nylon filament (diameter = 0.26 mm, Cinontech, Beijing, China) was inserted in the right CCA region and slowly advanced into the ICA (the length of nylon filament was about 18 mm) until it blocked the blood flow from the right middle cerebral artery. The reperfusion process was performed by gently removing the nylon filament after 90 minutes of obstruction. The wounds were then sutured and the rats allowed to recover. In the sham group, the CCA, ICA and ECA area were also exposed using ophthalmic scissors, and the incision region sutured without nylon filament insertion. In the MSCs-siRNA-CUEDC2+MCAO and MSCs-vector+ MCAO groups, 24 hours after the reperfusion phase, the SD rats were stereotactically injected with 2.0 × 10^6^ MSCs (siRNA-CUEDC2-MSCs and MSCs-vector) in a volume of 200 μL as previously reported [11]. After 14 days, the SD rats were sacrificed and the brain tissues analyzed as described below.

### Immunohistochemistry

CUEDC2 protein expression levels were confirmed by immunohistochemistry as previously described [41]. Briefly, 14 days after reperfusion, the brain tissues were obtained from the SD rats and fixed in 10% paraformaldehyde at 4° C for 24 hours. Brain tissues were then dehydrated using downgraded alcohol, embedded in paraffin, and sectioned into 5-μm thickness. The sectioned brain tissues were treated with 3% hydrogen peroxide (H_2_O_2_) to eliminate the activity of endogenous peroxidase, blocked with 10% goat serum in the buffer (Wellbio, Changsha, China) and incubated with CUEDC2 antibody (Abcom, Cambridge, MA). The nuclei were finally stained by Hematoxylin. The images of the rat cortical area were obtained microscopically (Olympus, Tokyo, Japan).

### Evaluation of neurological severity scores

The modified neurological severity score (mNSS) was used to determine the level of neurological deficit behavior in the rats [42]. Rats in the different groups were assessed on days: 0 (denoted before the MCAO operation), 3, 7, 14 and 21 (denoted after reperfusion time point). A neurological deficit score of 0 signified an absence of neurologic deficit behaviors, while higher scores signified severe neurological deficit behaviors. The details of the neurological behavior scores are presented in Supplementary Table 2.

### Determination of the infarct size with TTC staining

To confirm the cerebral infarct size after cerebral I/R in the MCAO model, brain tissues were stained with 2,3,5-triphenyltetrazolium chloride (TTC). Fourteen days after reperfusion, brain tissue slices (1 mm sickness) obtained from the various groups were treated with 1% TTC solution for 15 minutes at 37° C. The red stained area represented the normal brain tissues, while the unstained area represented the infarct brain tissues. The TTC staining images were obtained and the infarct volume quantified using the Image J software (https://imagej.en.softonic.com).

### Assessment of brain water content

The brain water content was calculated after cerebral I/R insult. Fourteen days after reperfusion, the whole brain was obtained and its wet weight measured. It was then dried at 100° C, and its dry weight measured again. The brain water content (%) = 100% × (wet brain weight – dry brain weigh)/wet brain weight.

### Hematoxylin-eosin (H&E) staining

H&E staining was done as previously reported [42] to observe the pathological alterations in the injured brain tissues. Briefly, 14 days after reperfusion, the brain tissues were paraffin-embedded and subsequently sliced into 5-μm sections. The sectioned brain tissues were then deparaffinized, and rehydrated. These sections were then stained with H&E (Well-BIO, Changsha, China) according to the manufacturer’s instructions.

### ELISA

The levels of TNF-α, IL-1β and IL-6 in brain tissues and cells were determined using the corresponding commercial assay kits, including Tumor Necrosis Factor-α Enzyme-Linked ImmunoSorbent Assay Kit, Interleukin-1β Enzyme-Linked ImmunoSorbent Assay Kit and Interleukin-6 Enzyme-Linked ImmunoSorbent Assay Kit (Beyond, Shanghai, China) according to the manufacturer’s instructions.

### Oxidative stress assay

OGD/R induced oxidative stress responses in the cells were assessed by measuring the secretion of reactive oxygen species (ROS), malondialdehyde (MDA), superoxide dismutase (SOD) and total antioxidant capacity (T-AOC) indices using the respective commercial kits, including Reactive Oxygen Species assay kit, Lipid Peroxidation MDA Assay Kit, Total Superoxide Dismutase Assay Kit and Total Antioxidant Capacity Assay Kit (Beyond, Shanghai, China) according to the manufacturer’s instructions.

### Statistical analyses

Statistical analyses were performed using GraphPad Prism 8.0 software (https://www.graphpad.com). After testing for normal distribution, data of two independent variables were analyzed using Student t-test. For three or more variables, one-way ANOVA was performed followed by post hoc analysis using Tukey's test. All data were presented as means ± standard deviations (SD). Differences between the mean values were considered significant at P value< 0.05.

### Data availability

The authors declare that all data supporting the findings of this study are available within the paper and its supplementary information files.

## Supplementary Material

Supplementary Figures

Supplementary Tables
