# Supplementary material for: CUEDC2 ablation enhances the efficacy of mesenchymal stem cells in ameliorating cerebral ischemia/reperfusion insult
Source: Aging (Albany NY). 2021 Jan 20;13(3):4335–56. doi: 10.18632/aging.202394 (PMC7906146; doi:10.18632/aging.202394)
Supplement: Supplementary Figures [file aging-13-202394-s001.pdf]

SUPPLEMENTARY FIGURES

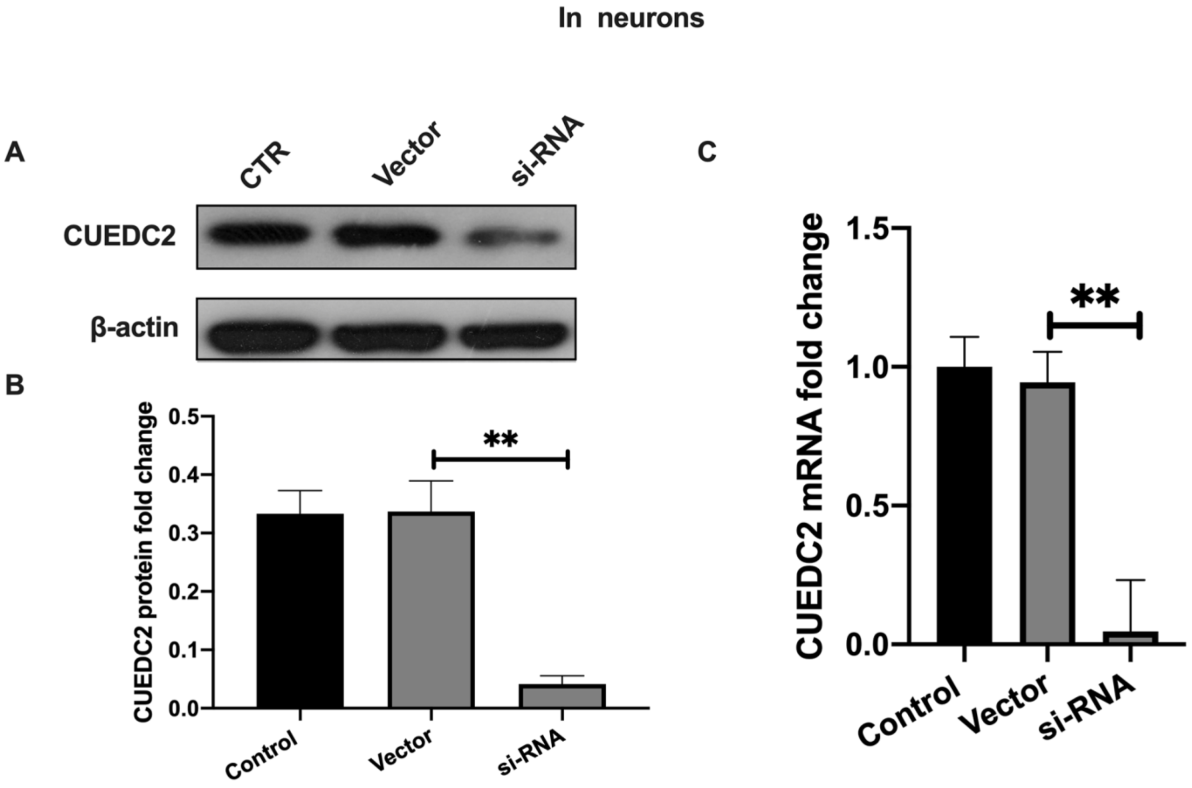

Supplementary Figure 1. Western blot and PCR analysis of CUEDC2 expression in CUEDC2 knocked-down neurons. (**A**, **B**) siRNA transfection efficiency determination in CUEDC2 protein and mRNA expression in knocked-down neurons. (**C**) siRNA transfection efficiency determination in CUEDC2 mRNA expression in knocked-down neurons.All data are presented as the mean value ±SD (n=3). Compared with the control group: **P<0.01; Compared with the control group and vector group: *P<0.05, **P<0.01.

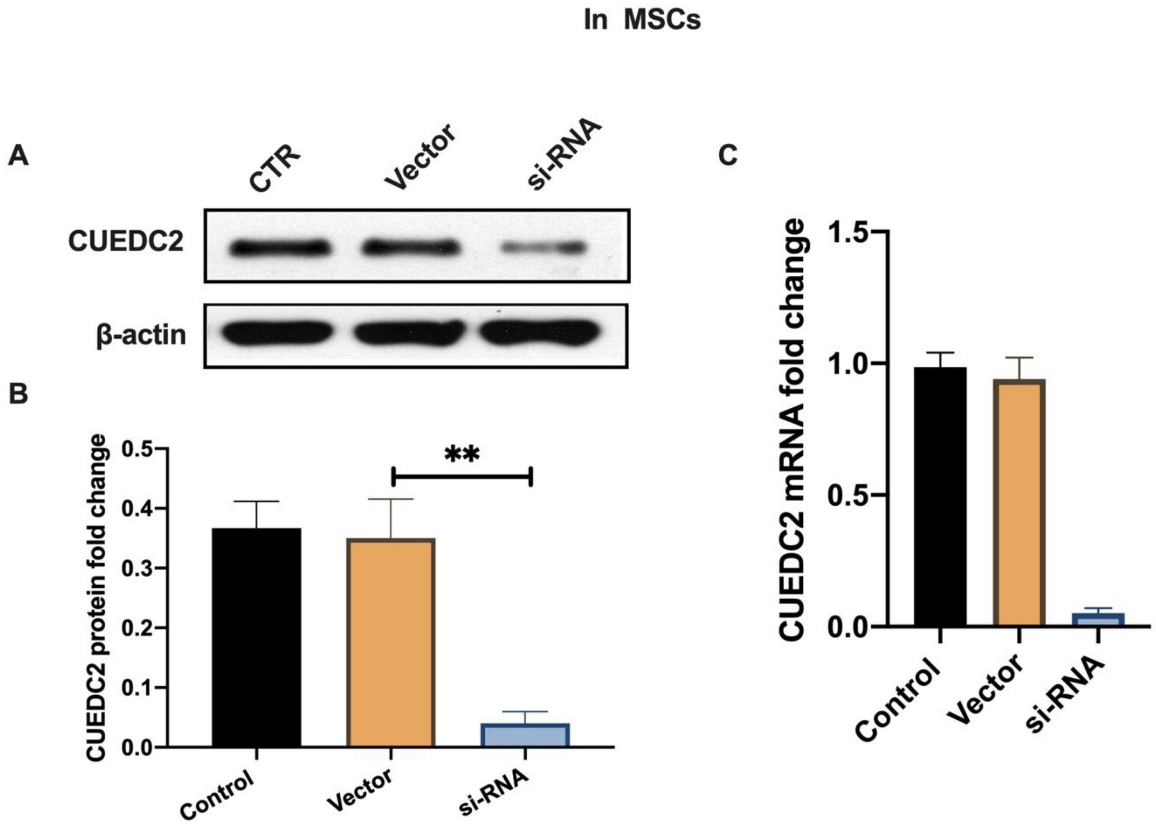

Supplementary Figure 2. Western blot and PCR analysis of CUEDC2 expression in CUEDC2 knocked-down MSCs. (**A**, **B**) siRNA transfection efficiency determination in CUEDC2 protein expression in knocked-down MSCs. (**C**) siRNA transfection efficiency determination in CUEDC2 mRNA expression in knocked-down MSCs. All data are presented as the mean value ±SD (n=3). Compared with the control group: **P<0.01; Compared with the control group and vector group: *P<0.05, **P<0.01.

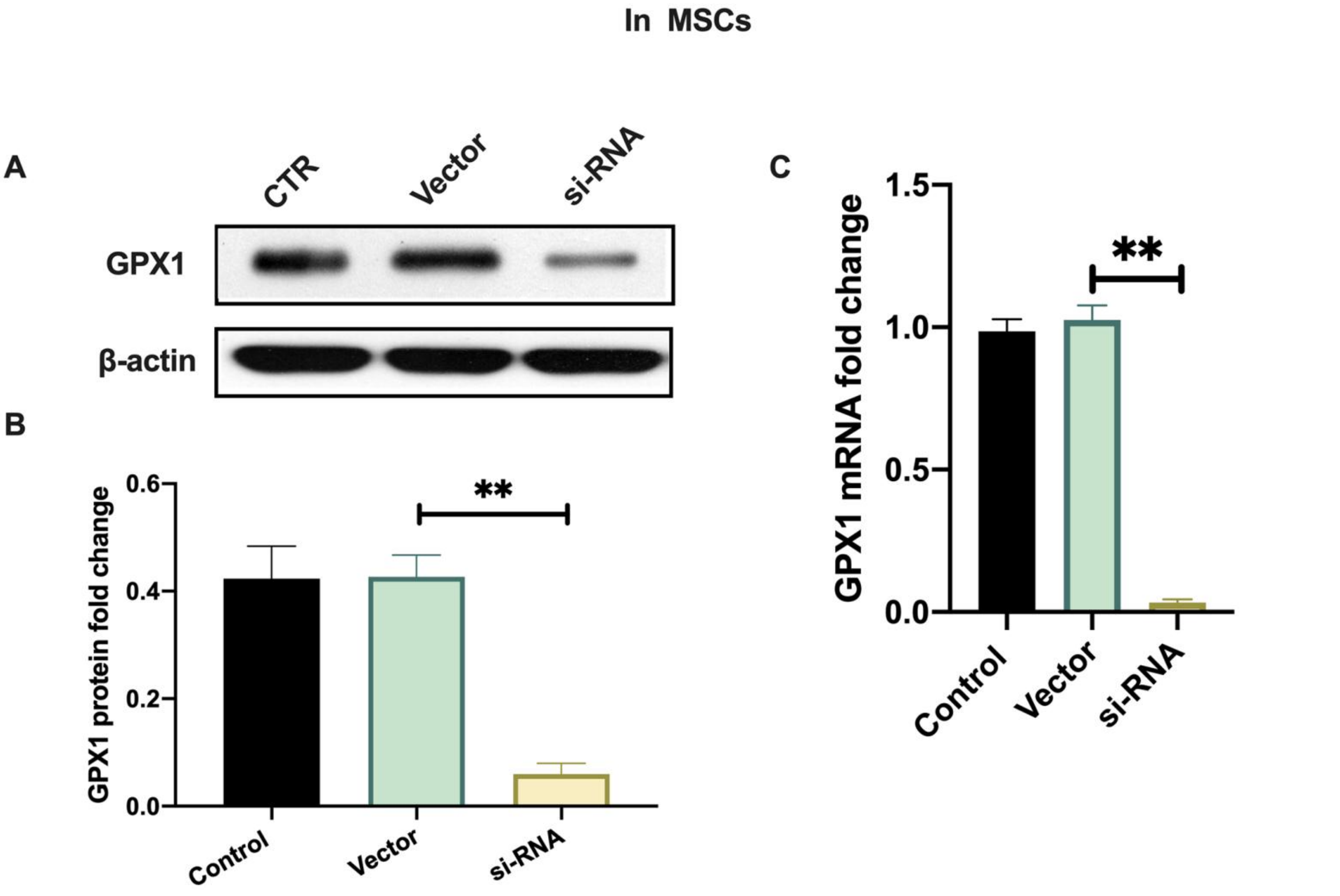

Supplementary Figure 3. Western blot and PCR analysis of GPX1 expression in GPX1 knocked-down MSCs. (**A**, **B**) siRNA transfection efficiency determination in GPX1 protein expression in knocked-down MSCs. (**C**) siRNA transfection efficiency determination in GPX1 mRNA expression in knocked-down MSCs. All data are presented as the mean value ±SD (n=3). Compared with the control group: **P<0.01; Compared with the control group and vector group: *P<0.05, **P<0.01.
