# Supplementary material for: CUEDC2 ablation enhances the efficacy of mesenchymal stem cells in ameliorating cerebral ischemia/reperfusion insult
Source: Aging (Albany NY). 2021 Jan 20;13(3):4335–56. doi: 10.18632/aging.202394 (PMC7906146; doi:10.18632/aging.202394)
Supplement: Supplementary Tables [file aging-13-202394-s002.pdf]

SUPPLEMENTARY TABLES

Supplementary Table 1. Primary and secondary antibodies.

Primary antibodies				
**Product name**	**Catalogue number**	**Host species**	**Concentration**	**Supplier**
CUEDC2	Ab79036	Rabbit	1:50	Abcam
GPX1	Ab22604	Rabbit	1:100	Abcam
caspase3	19677-1-AP	Rabbit	1:500	Proteintech
P65	10745-1-AP	Rabbit	1:2000	Proteintech
P-P65	#3033	Rabbit	1:1000	CST
β-actin	60008-1-Ig	Mouse	1:5000	Proteintech
Secondary antibodies				
HRP goat anti-mouse IgG	SA00001-1		1:5000	Proteintech
HRP goat anti-rabbit IgG	SA00001-2		1:6000	Proteintech

Supplementary Table 2. Modified neurological severity score grading system.

Points	Degree of injury
0	Normal
1-6	Mild injury
7-12	Moderate injury
13-18	Serious damage
18	Most severe neurological deficits
